# Genomic Approach to Understand the Association of DNA Repair with Longevity and Healthy Aging Using Genomic Databases of Oldest-Old Population

**DOI:** 10.1155/2018/2984730

**Published:** 2018-05-03

**Authors:** Yeo Jin Kim, Hyun Soo Kim, Young Rok Seo

**Affiliations:** ^1^Department of Life Science, Dongguk University Biomedi Campus, 32 Dongguk-ro, Ilsandong-gu, Goyang-si, Gyeonggi-do 10326, Republic of Korea; ^2^Institute of Environmental Medicine, Dongguk University Biomedi Campus, 32 Dongguk-ro, Ilsandong-gu, Goyang-si, Gyeonggi-do 10326, Republic of Korea; ^3^The Jackson Laboratory for Genomic Medicine, 10 Discovery Drive, Farmington, CT 06032, USA

## Abstract

Aged population is increasing worldwide due to the aging process that is inevitable. Accordingly, longevity and healthy aging have been spotlighted to promote social contribution of aged population. Many studies in the past few decades have reported the process of aging and longevity, emphasizing the importance of maintaining genomic stability in exceptionally long-lived population. Underlying reason of longevity remains unclear due to its complexity involving multiple factors. With advances in sequencing technology and human genome-associated approaches, studies based on population-based genomic studies are increasing. In this review, we summarize recent longevity and healthy aging studies of human population focusing on DNA repair as a major factor in maintaining genome integrity. To keep pace with recent growth in genomic research, aging- and longevity-associated genomic databases are also briefly introduced. To suggest novel approaches to investigate longevity-associated genetic variants related to DNA repair using genomic databases, gene set analysis was conducted, focusing on DNA repair- and longevity-associated genes. Their biological networks were additionally analyzed to grasp major factors containing genetic variants of human longevity and healthy aging in DNA repair mechanisms. In summary, this review emphasizes DNA repair activity in human longevity and suggests approach to conduct DNA repair-associated genomic study on human healthy aging.

## 1. Introduction

Aging is an inevitable process in human life. Many countries are rapidly transitioning to an aging society due to increasing life expectancy and advanced medical supports [1–3]. Over the last few decades, the advent of aging society is considered a crucial issue that may cause future decline in productivity of community [1, 4]. Many researchers have recently warned that urban environmental pollutants can cause physiological weakness and increase the risk of premature aging or chronic diseases in the elderly population [5–9]. Thus, interest in antiaging and healthy longevity is constantly increasing. “Active aging” or “successful aging” has been spotlighted as a strategy to promote social contribution of the elderly [10]. The definition of successful aging remains controversial. However, its main point is to live a healthy life in physical, cognitive, and biomedical aspects [10–12]. Although many studies have dealt with the topic of aging in the past, it is too complex to clearly understand fundamental causes of the aging process.

Longevity is usually defined as living until life expectancy that is typically over 85 years old. Exceptional longevity such as centenarians is considered when one is more than 95 years old with a healthy life [10, 13]. Several researchers have emphasized the importance of in-depth studies on longevity to cope with an aging society [14, 15] because such studies could suggest various biomedical clues for living a long and healthy life. Oldest-old individuals, often centenarians, represent an adequate model to investigate the complex phenotype of healthy longevity. Among enormous population-based studies on centenarians, one major focus is on people with exceptionally long lives without functional impairment [10, 16–21]. Several landmark studies on healthy centenarians have found that the progression of major diseases such as cancer, cardiovascular disease, and stroke is delayed in the oldest group compared to that in the other younger or same-aged control groups, suggesting a substantial relationship between healthspan and longevity [10, 20, 22].

Although successful longevity traits are modulated by various factors, such as environmental, behavioral, and/or endogenous causes, genetic factor might be a major factor that contributes to healthy aging. Within the past few decades, many researchers have tried to identify longevity-associated genes using diverse species, ranging from less complex organisms to higher organisms [18, 23–26]. With development in genomic technology, genetic factors associated with longevity have been suggested in human population studies and human genome-wide association studies [18, 21, 27]. It has been found that variants of APOE and FOXO3A are highly associated with longevity. This finding has been consistently replicated in many different population-based studies [21, 28–30]. Despite the complexity of healthy longevity in human due to various influences, genetic factors are thought to be exceedingly important to understand the genetic basis of longevity. Accordingly, many studies have investigated various genetic factors, including nuclear genomic variants, mitochondrial variants, telomere, and epigenetics, to elucidate the substantial contribution of genetic factors to longevity [31–34].

Accumulation of DNA damage is associated with functional decline in the aging process [35–37]. Thus, maintenance of genomic integrity might be a crucial factor for healthy life and longevity. Genome instability generally increases with age. DNA repair machineries control genome stability [38]. Previous studies on centenarian have shown that oldest-old population have enhanced DNA repair activity with significant lower frequency in genomic and cellular damage compared to their younger counterparts [35, 39, 40]. Thus, DNA repair plays an important role in understanding exceptionally long-lived individuals.

In this review, we focus on major DNA repair machineries associated with longevity. We also explored longevity-associated population studies using genome-wide approaches. With brief introductions of genomic databases in aging and longevity field, ample genomic resources of normal long-lived human population were utilized for DNA repair-focused approach. Herein, we suggest a new aspect of longevity study to investigate the complex interplay between DNA repair and longevity by processing human genetic variations based on previous studies, providing a brief interpretation of their molecular networks. This review not only provides an overview of the importance of DNA repair mechanism in longevity but also suggests a novel approach to select candidate genes associated with healthy aging in human.

## 2. Healthy Traits of Long-Lived Population

As concerns about longevity increase, many research studies have investigated longevity using model organisms to understand the association between genetic contribution and lifespan [23, 24, 26, 41–43]. However, human lifespan is too complex to clearly elucidate its biological and sociocultural factors. Therefore, many studies on human longevity have been conducted epidemiologically by comparing populations divided by age [44–47]. Recently, older population with good health and longevity has been investigated to characterize healthy aging phenotypes and differences compared to those with same age or younger to provide better public health care [48–51]. Nolen et al. have published a comprehensive review on cancer prevalence in the oldest-old population and found that centenarians and the oldest-old have lower risk of cancer [50]. In Japan, where there is a relatively high population of centenarians, the Okinawa Centenarian Study, the world's longest-running population-based study of centenarians, has been performed to understand the contribution of genetic and environmental factors to exceptional longevity [18]. Interestingly, these studies commonly concluded that not all elder people showed higher degree of age-associated disorders. In fact, long-lived individuals with inherited predisposition and their offspring showed beneficial profiles of major disabilities [18, 48–50, 52, 53]. However, understanding about the effect of genetic factors on longevity is still limited. Novel gene and/or genetic variations and contribution of different aspects to longevity need to be determined in the future.

Enhanced DNA repair capacity is thought to be a crucial factor for healthy longevity based on previous studies using oldest-old population [40, 54]. Evidence for improved DNA repair system that leads to delayed aging has been accumulated based on several human population studies [55–57]. Indeed, the frequency of DNA damages such as cytogenic aberrations and micronuclei is significantly lower in the oldest age group than that in the other groups, suggesting more genomic stability in the oldest-old population [58]. However, Chevanne et al. [40] have reported that DNA repair capacity in centenarians is similar to that in young generations. In accordance to these findings, the importance of DNA repair activity in longevity needs to be clarified to elucidate factors associated with longevity.

## 3. DNA Repair and Longevity

Disturbance of genome integrity is commonly known as a staple factor in the etiology of age-related cellular dysfunction and pathogenesis, although a plethora of extrinsic and intrinsic factors can also threaten genome stability. Accumulated DNA damage can lead to cellular dysfunction, cell death, and carcinogenesis. Generally, DNA repair mechanisms in cellular protection system can rescue various cytotoxic and mutagenic lesions to maintain DNA integrity. Accordingly, studies on the association between DNA repair mechanism and aging are increasing. In this review, we only focused on DNA double-strand break repair, base excision repair (BER), and nucleotide excision repair (NER) associated with aging and longevity in terms of maintaining genome integrity. Although there are many kinds of DNA repair mechanisms to prevent genomic instability, other pathways have been more related to diseases such as cancer and disorders other than aging [59–62].

Age-related increase of DNA double-strand breaks is consistently considered as a genetic blueprint of progeroid syndromes because DNA double-strand breaks cause the most deleterious damage to DNA [63–65]. Major repair pathways for DNA double-strand breaks are homologous recombination (HR) and nonhomologous end joining (NHEJ). HR uses undamaged sister chromatid as template during cell division. It is an error-free pathway [66]. NHEJ occurs even in G1 phase of the cell cycle where sister chromatid does not exist. It can join the ends of a double-stand break without a template [67]. Several studies have shown that DNA double-strand break repair is reduced in the aging population [63, 68, 69]. Many proteins involved in the NHEJ process need to maintain telomeres. Ku70, Ku80, DNA-PK_CS_, WRN, and PARP1 are key proteins of genome integrity [70–73]. Deficiency of these proteins induces premature aging and age-associated disorders [74–76]. Recently, it has been found that SIRT6, one longevity gene, is involved in DNA double-strand break repair by recruiting PARP1 to damaged DNA region [77].

One major hypothesis on aging is that exposure to reactive oxygen species (ROS) is increased over the lifespan [63, 69, 78]. The production of ROS can be induced by multiple extrinsic and intrinsic factors. It causes various kinds of DNA damage, including apurinic/apyrimidinic sites due to DNA base lose, single-strand break, and double-strand break [79, 80]. Accumulated DNA damages due to ROS frequently lead to cellular dysfunction, a known consequence of chronic oxidative stress with aging [78]. Several defense mechanisms, including DNA repair machinery, can cope with the threat of ROS [81]. BER predominantly corrects oxidative lesions [82]. Indeed, many subunits such as APE1, PCNA, and HSP70 related to BER pathway are involved in the defense mechanism against cellular oxidative stress, including DNA repair [82]. Many studies have shown the association between BER and aging. For example, BER capacity is significantly decreased in brain and liver tissues of old mice [83]. Many studies have also reported that the decline of major components (pol*β*, pol*γ*, APE1, and Sirt6) of BER pathway is associated with aging [84–89]. Interestingly, deficiency of APE1, a vital element of BER initiation, leads to telomere dysfunction and segregation, suggesting that BER plays a role in aging through telomere protection [90, 91].

NER, another type of DNA repair pathway, copes with a wide range of lesions that distort the double helix structure of DNA [81]. DNA bulky damages recognized by NER can cause premature aging and/or cancer [92, 93]. NER is subdivided into global genome NER and transcription-coupled NER depending on where it occurs, covering lesions that can be detected by NER subunits [60, 94]. Some NER proteins are thought to be important factors in the aging process due to their direct association with progeroid syndromes such as trichothiodystrophy (TTD), Cockayne syndrome (CS), and xeroderma pigmentosum (XP) [95–98]. A point mutation at different sites in XPD gene can trigger TTD or CS [96]. A defect in XP gene family (XPA-XPG) induces XP. The patient with such defect has shown dramatically accelerated skin aging [99]. Although whether decline of NER efficiency is associated with aging remains controversial [100–102], defect in NER machinery virtually provokes age-related pathology and premature aging. Hermetic effects on this aspect supports the crucial role of NER in healthy aging through conserved pathway [103–105]. A prominent mechanism of cellular protective responses is regulation of IGF-1 signaling that leads to somatotropic attenuation by RNA polymerase II stalling. Interestingly, this prosurvival response was commonly found in naturally aged, progeroid, and long-lived mutant mice [106–108]. However, the mechanism eventually enhances longevity assurance in wild type, while it has severe consequences in NER defects [104, 105]. In this regard, the modulation of DNA damage is thought to be a more significant factor with a prosurvival harbor [107]. Other intrinsic or extrinsic factors, of course, should have to be considered for elucidating this complicated process. Thus, understanding longevity in terms of DNA repair is crucial in the aspect of genome integrity preservation. The complex interplay between DNA repair and longevity remains unclear.

## 4. Genomic Resources for Understanding Aging and Longevity

Although longevity is a multifactorial process, genomic approaches can be used to elucidate biological aspects of longevity by identifying standardized parameters such as biomarkers [109]. With development of next-generation sequencing, a large number of long-lived individuals have been studied to obtain their specific genomic information such as single nucleotide polymorphisms, copy number variations, transcriptomics, and epigenomics [110–114]. Although disease-susceptibility alleles are well characterized in genome-wide association study (GWAS) catalog by the National Human Genome Research Institute, research data for illustrating low frequency of disease alleles in exceptional longevity are limited or controversial [115]. Up to date, APOE and FOXO3A have been consistently suggested as well-described candidate genes in human longevity by various cross-sectional studies [21, 116, 117]. Furthermore, joint roles of genetic variants and phenotypes in longevity have been suggested to improve our understanding on aging and longevity [48]. Pathway-based candidate gene studies have been performed to encompass their molecular and biological networks in longevity [118–121]. However, their roles in longevity remain controversial.

Based on exponentially accumulated data, major aging research groups have started global interdisciplinary collaboration to share large scale genomic resources obtained from sequencing data [122]. Human Ageing Genomic Resources (HAGR; http://genomics.senescence.info) provides in-depth information about the biology and genetics of aging [123]. HAGR now includes six core databases: GenAge, AnAge, GenDR, DrugAge, and LongevityMap. GenAge contains benchmark database of genes associated with aging. It is now subdivided into two: (1) potential aging-related genes in human and (2) lifespan-associated genes in model organisms [124]. AnAge is a database of aging and longevity in animals for comparative and evolutionary studies in this field [124]. Since there are many theories and factors of aging and longevity, HAGR has been expanded. It now has new categories to deal with different aspects of this issue. GenDR is focused on dietary restriction. DrugAge is a database of life-extending drugs in model organisms. CellAge is a very recent database to support overall cellular longevity study [125]. LongevityMap is an inclusive database based on genomic studies of human longevity and healthy aging, excluding long-lived individuals who have unhealthy traits such as disease, disorder, and/or dysfunction [126]. Utilizing these open source data may aid biogerontologists to interpret human aging and longevity in diverse aspects of the complex process involved in aging and longevity.

## 5. DNA Repair and Longevity-Associated Genetic Variation

With valuable longevity population data in longevity databases, we investigated healthy longevity-associated genetic variations in terms of major elements of DNA repair mechanism. We focused on people with normal phenotype in elder population. Data of human genetic variants associated with longevity were retrieved from LongevityMap. Contents of enormous studies on human longevity and healthy aging ranging from cross-sectional investigations to extreme longevity are curated in LongevityMap [126]. We trimmed these data by their significance in association with longevity. To elucidate correlations between human healthy longevity and DNA repair in the aspect of genetic variants, we collected genes associated with canonical DNA repair mechanisms from well-reviewed publications. DNA repair-associated genes were obtained through search using keywords such as NER, BER, NHEJ, HR, and MMR in human-oriented samples. Gene sets that had significant association with longevity and DNA repair were analyzed to identify common genes in these two groups. As a result, 16 genes were obtained, including key factors of DNA repair mechanism such as TP53, ATM, WRN, and POLB (Table 1). These results should be cautiously grasped. For instance, in case of rs1042522 on TP53 gene, two different population studies suggested opposite interpretations on the same SNP [127, 128]. However, the two studies also described common cellular effects of each allele as well. This may be due to differentially designed population studies (cross-sectional versus prospective follow-up). Their advantages and pitfalls in each methodological strategy must be considered seriously to understand population studies, especially, for aging and longevity, because these are very complex and multifactorial processes. Therefore, complicated interactions instead of a single factor should be taken into account. In addition, integrative approach should be used to understand aging and longevity.

## 6. Complex Interplay on DNA Repair Mechanism

**Figure 1:**
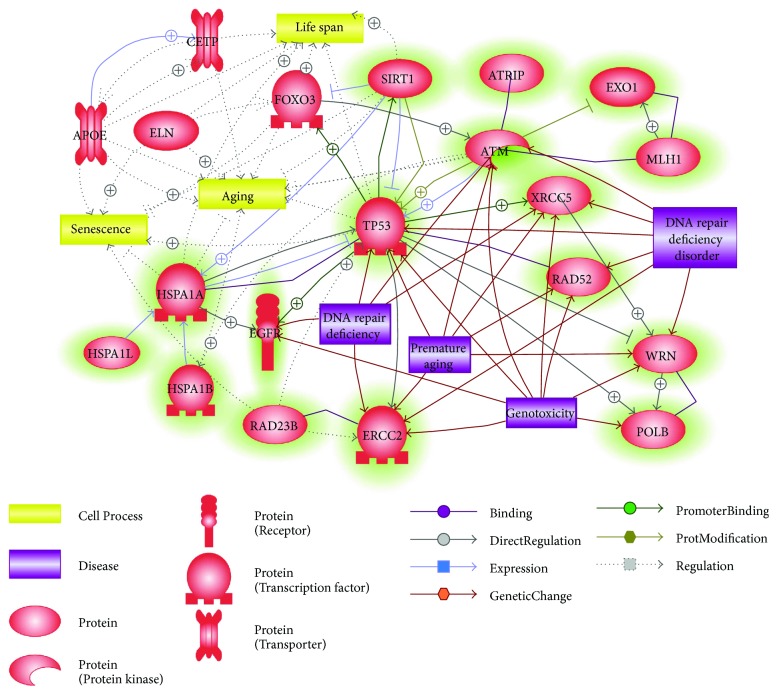
Direct networks among genes obtained by comparison of gene sets associated with DNA repair and human longevity. The analysis of molecular and biological networks was conducted by using Pathway Studio Web (version 11.4.0.8). Green highlighted entities indicate 16 common genes obtained by gene set comparison. These networks were built with careful curation considering the number of reference (>10) and their correlation with longevity, aging, and DNA repair.

To interpret the meaning of these common genes, biological network analysis was conducted using Pathway Studio, a text mining-based pathway analysis program. Recently, analyzing molecular network is considered a more critical part than just detecting alteration of DNA sequence and/or gene expression to understand difference in phenotype. Various tools have been developed to conduct network analysis for genes of interest due to advance in bioinformatics industry and accumulating research products. Pathway Studio as a commercial software for biological pathway analysis can navigate related biological processes using data mining interface [129]. In this review, we explored interacting networks of these 16 common genes to elucidate the role of DNA repair in longevity using Pathway Studio. These genes were initially analyzed for their direct interactions and correlations with lifespan-associated genes and cell processes (Figure 1). Many of these genes are known as genetic parameters of genomic instability and premature aging. According to results of our network analysis, TP53, ATM, and SIRT1 were the top three elements with high number of connections with others, suggesting that their genetic variants might be considered as key nodes to elucidate genetic contribution of major DNA repair factors to longevity and healthy aging.

**Figure 2:**
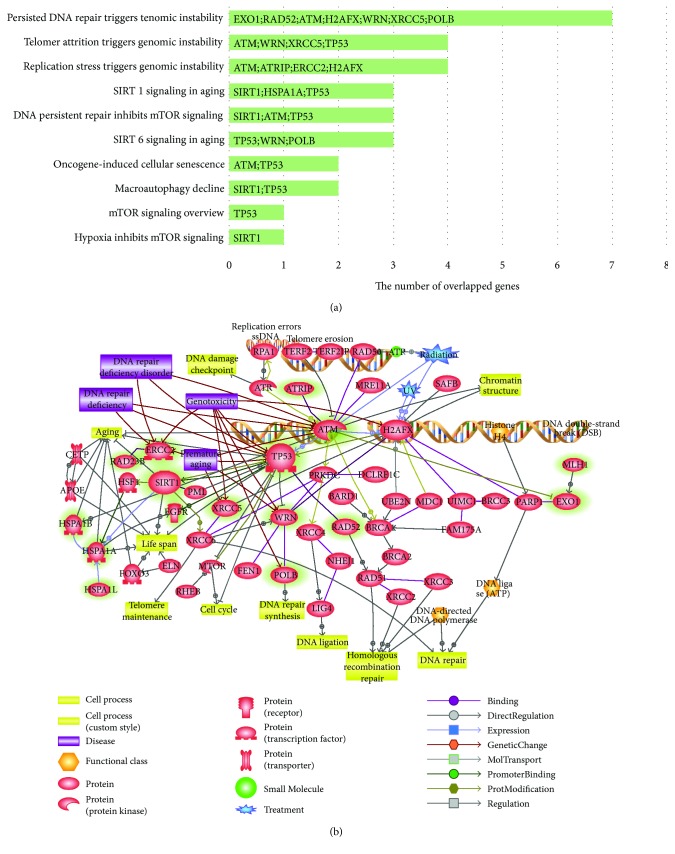
Major enriched networks of common genes and the top ranked pathway suggesting key contributors to longevity in aspect of DNA repair. (a) Pathways and ontologies enriched in these genes shown in a bar graph. *x*-axis indicates the number of overlapped genes with elements of each pathway/ontology while *y*-axis shows the name of statistically meaningful pathways/ontologies (*p* value < 0.05). (b) The most enriched pathway, “persisted DNA repair triggers genomic instability,” and direct pathway analyzed previously were combined using Pathway Studio Web (version 11.4.0.8) to explore major genes including candidate longevity-associated loci in DNA repair to provide better visualization. Green highlighted entities indicate common genes collected by gene set comparison.

We also conducted gene set enrichment analysis using the Pathway Studio software to explore which pathways and ontologies might be mostly involved in these common genes associated with DNA repair and longevity. Statistical enrichment in this gene set was collected. We curated the result by a *p* value of less than 0.05 (Figure 2(a)). Approximately half of these 16 common genes had overlapped biological function, namely, “persisted DNA repair triggers genomic instability.” For better visualization, pathways of biological function derived from this software and those of direct network from our analysis were combined (Figure 2(b)). The results showed that ATM and TP53 played a major role in DNA repair by detecting DNA damage and modulating downstream DNA repair machineries. Although further meticulous study is needed to confirm their roles in longevity, longevity-associated human genetic variants in TP53, ATM, and SIRT1 are worth considering to identify potential key factors and understand the linkage of DNA repair to longevity and healthy aging.

## 7. Conclusions

Although the importance of genomic stability in longevity is continuously discussed [35, 130, 131], studies using genomic and molecular approaches to understand genetic variations of extremely old population in the aspect of DNA repair are limited. In this review, we focused on DNA repair mechanisms associated with longevity and healthy aging to elucidate their effects on the aging process. As reported in many studies, this review also emphasized the role of DNA repair in maintaining genome integrity as a crucial factor for healthy longevity. With enormous resources of human longevity population that are freely available online based on NGS studies, DNA repair-focused approach is useful for identifying the association of genes with longevity by integrated network analysis. This research approach could be ideal and valuable for handling genomic data. The present review may provide a clue to utilize genomic databases to elucidate contribution of genetic factors to longevity in many different aspects. Although we only dealt with DNA repair associated with longevity, comprehensive data from lifestyles should be considered to better understand the process of healthy aging. It might lead to the development of personalized antiaging strategy.

## Figures and Tables

**Table 1:** Summary of DNA repair-associated genes with significant genomic variants in longevity.

Genes	Variations	Region	Populations	References
ATM	rs189037	5′-UTR	Chinese (Han)	[132]
			Italian	[133]

ATRIP	rs9876781	Upstream	American (Caucasian)	[134]

EGFR	rs2072454	Exon (synonymous)	Korean	[135]
	rs2293347	Exon (synonymous)		
	rs3807362	3′-UTR		
	rs884225	3′-UTR		

ERCC2	Lys751Gln^§^	Missense (stop-gain)	Polish	[136]
EXO1	rs1776180	Upstream	German	[137]
	rs735943	Exon (missense; H/R)		
	rs4149965	Exon (missense; V/M or V/L)		

HSPA1A	−110A/C	Upstream^§§^	Italian (Southern)	[138]
			Danish	[139–141]
	G190C	^§§^	Chinese (Uighur in Xinjiang)	[142]

HSPA1B	A1267G	^§§^	Danish	[141]
			Chinese (Uighur in Xinjiang)	[142]

HSPA1L	T2437C	^§§^	Danish	[141]
			Chinese (Uighur in Xinjiang)	[142]

MLH1	C670, A676, T1172	^§§^	Korean	[143]
	rs13320360	Intron	Danish	[119]

POLB	rs2953983	Intron	Danish	[119]

RAD23B	rs1805329	Exon (missense; A/V)	Danish	[119]

RAD52	rs11571461	Intron	Danish	[119]

SIRT1	rs3758391	Upstream	Chinese (Han)	[144]
	rs4746720	3′-UTR		
	rs7896005	Intron	American (Caucasian)	[145]
	rs12778366	Upstream	Dutch	[146]

TP53	rs1042522	Exon (missense; P/R or P/H)	Danish	[127]
			Italian (Central)	[128]
	rs9616906	Upstream	American (Caucasian)	[134]

WRN	rs13251813	Intron	Danish	[119]

XRCC5	rs705649	Intron	Danish	[119]

^§^Variation in amino acid; ^§§^studies on haplotype analysis.
